# Availability of an RFID Object-Identification System in IoT Environments

**DOI:** 10.3390/s21186220

**Published:** 2021-09-16

**Authors:** Cosmina Corches, Mihai Daraban, Liviu Miclea

**Affiliations:** 1 Department of Automation, Faculty of Automation and Computer Science, Technical University of Cluj-Napoca, 400114 Cluj-Napoca, Romania; cosmina.corches@aut.utcluj.ro; 2 Applied Electronics Department, Faculty of Electronics, Telecommunications and Information Technology, Technical University of Cluj-Napoca, 400114 Cluj-Napoca, Romania; mihai.daraban@ael.utcluj.ro

**Keywords:** RFID, edge computing, IoT, SPN, dependability, availability

## Abstract

Through the latest technological and conceptual developments, the centralized cloud-computing approach has moved to structures such as edge, fog, and the Internet of Things (IoT), approaching end users. As mobile network operators (MNOs) implement the new 5G standards, enterprise computing function shifts to the edge. In parallel to interconnection topics, there is the issue of global impact over the environment. The idea is to develop IoT devices to eliminate the greenhouse effect of current applications. Radio-frequency identification (RFID) is the technology that has this potential, and it can be used in applications ranging from identifying a person to granting access in a building. Past studies have focused on how to improve RFID communication or to achieve maximal throughput. However, for many applications, system latency and availability are critical aspects. This paper examines, through stochastic Petri nets (SPNs), the availability, dependability, and latency of an object-identification system that uses RFID tags. Through the performed analysis, the optimal balance between latency and throughput was identified. Analyzing multiple communication scenarios revealed the availability of such a system when deployed at the edge layer.

## 1. Introduction

Radio-frequency identification (RFID) technology is increasingly common around us, being used in package identification, healthcare, supply-chain management, controlling access within a building, and detecting objects or a person’s location during context build [1,2,3,4,5,6,7]. From a system point of view, an RFID network consists of RFID tags, antenna, and reader, and the software component responsible with managing the information. The RFID reader retrieves data from the RFID tags and passes them over to the managing software for storage and interpretation [5,8,9]. What sets RFID technology apart from other wireless technologies is the simplicity of producing the tags, which can be active or passive [9]. Compared to other traditional technologies, for example, bare-code reading, RFID has the advantage of allowing for the simultaneous reading of multiple tags that do not need line-of-sight alignments [6]. Passive RFID tags have gained momentum in the current context of reducing energy consumption and environmental impact [10,11,12], and in the current development of the Internet of Things (IoT) [13]. Although active RFID tags have advantages in terms of the communication distance and available resources, the presence of batteries implies higher production costs and a higher degree of pollution because of their manufacturing process.

The main objective of RFID-based applications is successful object identification [14]. Even though algorithms for advanced image processing have evolved, low environmental light or image stabilization can affect the process [7], which is why RFID can act as a complementary technology to improve object detection. For example, image processing can be very useful in monitoring patients taking their medication [15]. However, RFID can be very useful in monitoring the type of drug that was taken by placing RFID tags on medicine bottles [5,6,15], thus helping to increase confidence in the obtained identification results.

Another advantage of RFID technology is the ease with which it can integrate into the cloud–fog–edge architecture. In the current context, with the advent of IoT devices, the trend is to move advanced processing to the cloud or fog layer. Meanwhile, at the edge layer, processes that require fewer resources are kept. Such sharing solves the problems of response time and data congestion that were common when only using the cloud layer [16].

Through the cloud–fog–edge architecture, the object-identification process can be shared, such that advanced image processing can be performed in the cloud or fog layer, while RFID-based object detection is performed at the edge layer. The advantage of such a system allows for real-time location data, while more complex context-related information resulting from cloud-layer processing is provided later. Even if RFID has many advantages, RFID tag collision is a problem in a RFID object-identification system [14].

This paper analyzes the availability of an object-identification system through RFID technology, analyzing the entire communication process that takes place at the edge layer. We focus on the response time of a single RFID reader surrounded by multiple tags during the identification process to identify the optimal system cost as the time length of a frame inventory per tag. Compared to other papers, we also consider the probability of encountering event cost during system implementation. The RFID frame size is based on the ISO15963 standard, which consists of 16 slots and uses Frame Slotted Aloha (FSA) to solve collision [17].

There are studies in the literature that analyze identifying objects through RFID technology, but they focus on determining the maximal throughput that can be obtained on the basis of the number of slots present in a query frame and the number of tags present within the range of the RFID reader’s antenna [18,19,20,21]. Depending on the type of analysis, throughput can be either the number of queries per second or the number of tags identified in an amount of time.

This paper does not contradict the current research but highlights the probability of achieving different throughput values. Even if the current paper uses the equations describing the probability associated with the FSA protocol, by using stochastic Petri nets (SPNs), we recalculate, at each new frame inquiry, the probabilities associated with tag identification. The RFID identification process is usually described from a probability point of view as the number of slots in a frame and the number of tags in the system. However, after a frame inquiry, there can be several identified tags and several pairs of RFID tags that are in collision. Hence, more frame inquiries are needed to solve the collisions.

Through the proposed SPN models, we computed what the total number of frames is that is required to identify the system tags (resulting in a cost value), and what the probability for such an event is. The proposed model allows for recomputing at each frame the probabilities associated to the three events that might appear in a frame inquiry: one tag per slot, slot without a response, and a slot with collision. In a real-time system, it is important that data are provided in a timely manner, and that the process is predictable. In this paper, analyses are carried out on the probability of obtaining a certain cost.

## 2. RFID Collision Analysis

**Figure 1:**
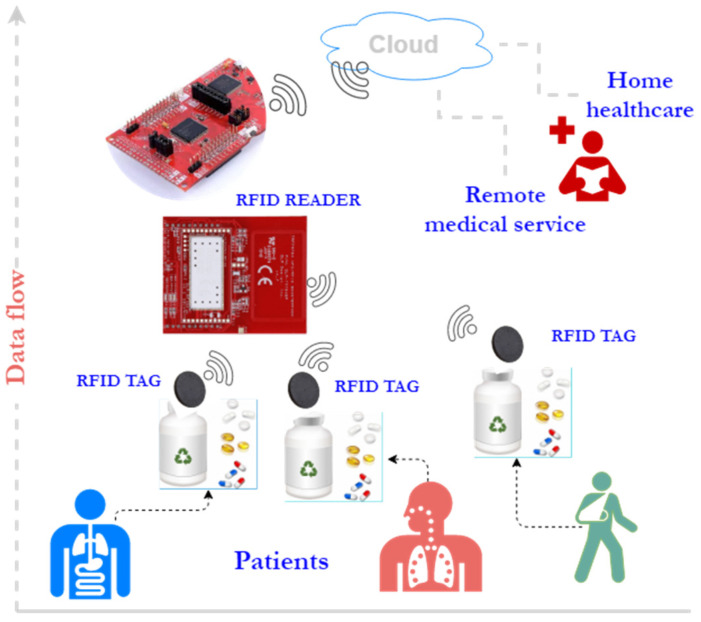
Patient-monitoring architecture.

An example where advanced image processing can be used with the identification of objects through RFID is the monitoring of drug administration to the elderly [1,15]. Image processing monitors gestures associated with the drug-administration process, while the association of RFID tags to the pack of medicines allows for the identification of the administered treatment. The monitoring process can be performed with video cameras and a network of RFID antennas, but this is an expensive setup. An alternative is to use a robot capable of capturing and transmitting video information in the cloud and to be equipped with an RFID reader (Figure 1).

**Figure 2:**
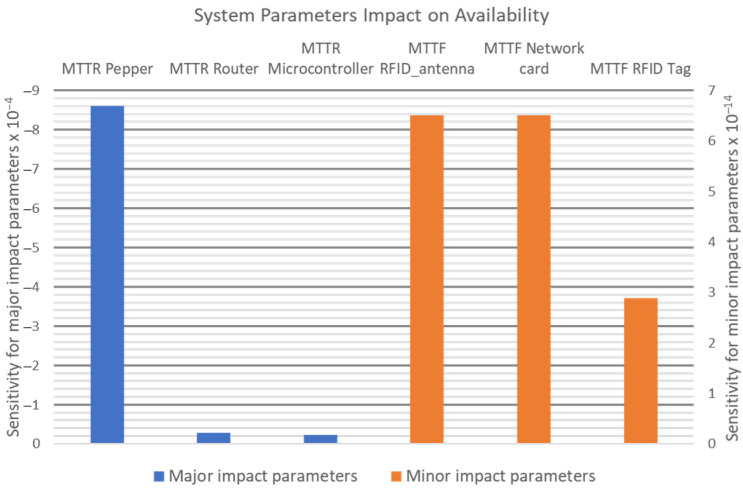
Radio-frequency identification (RFID) object identification system—major and minor parameters’ impact on system availability.

Such a setup was proposed in [22], where besides the Pepper robot, a setup was added comprising a microcontroller and RFID reader. Following the analysis of the setup through reliability block diagrams (RBDs), the components that had the greatest impact on the availability of the object-identification system were identified. Figure 2 shows the parameters (i.e., mean time to failure (MTTF) and mean time to repair (MTTR)) that have a major impact versus ones with minor impact on system availability.

Although the analysis of a system in terms of dependability is relevant, within an object-identification system, the time during which the information is transmitted is also important. To determine the required time to detect the unique identifiers (UIDs) associated to each tag in the vicinity of the RFID reader, SPN-based analysis is performed.

### 2.1. RFID Collision Analysis through Stochastic Petri Nets

**Figure 3:**
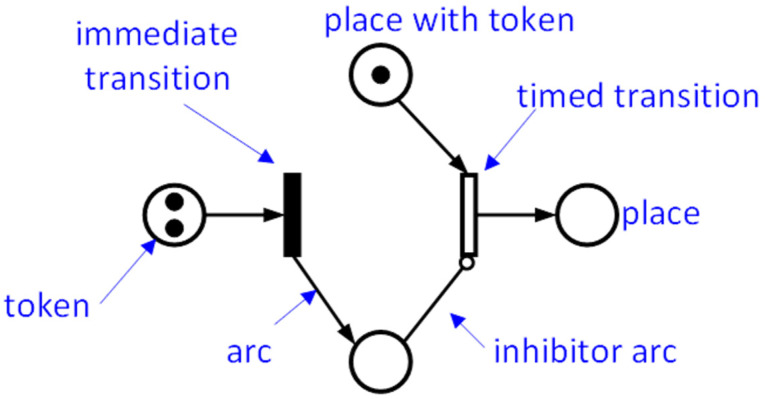
Basic graphical symbols of stochastic Petri nets (SPNs).

Determining the availability of a system, including the relationships between operating conditions and events occurring during operation, it is necessary to use Petri nodes. Initially, the analytical model based on Petri nodes does not contain the time component, but it is added later, with it becoming an SPN. By performing the analysis, it is possible to model the state of the components that comprise the system, so that the state of the system derives from the state of its components, which are no longer explicitly expressed [23]. A system is described through the SPN using symbols that describe the events, transitions, and states of the system (Figure 3). As the SPN model takes into account the events during the RFID anticollision algorithm, it allows for an improved estimation of the time (i.e., as number of inquire frames) needed to identify the RFID tags. In this paper, the SPN is used to model the availability of an RFID system (RFID tags, antenna, and reader, and robot software component responsible with managing the information), and to find the optimal number of tags that could be identified through the smallest number of inquiry frames.

**Figure 4:**
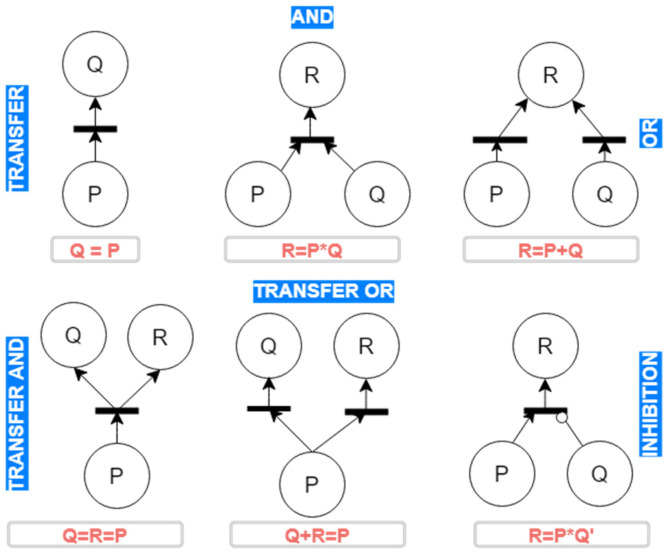
Logic relations for Petri nets.

For a transition to fire, inputs from events must meet the imposed condition by the transition [23,24]. When a transition is fired, it absorbs one token from the connected places as input. Following this process, tokens are produced to the exit events (places). Figure 4 illustrates the transitions that can be used in SPN analysis.

Figure 4 shows direct input places of the specified or conditional type. An example of a specified input place is the AND logic relation, in which an applied token to the input propagates to two events. The conditional type (OR-type propagation) offers several token propagation paths, which can lead the system to different operating situations. The choice of a particular path depends on the probabilities of events, the additional actions in the system, or the conditions imposed on the events.

Besides immediate and time-conditioned transitions (having a constant trigger duration), there are also stochastic transitions. The latter are useful in modeling processes with random trigger times.

The previous descriptions show that SPN is a technique that can be used both in analyzing reliability and monitoring the failure rate, and to observe the system’s dynamic behavior. This helps in tracking the spread of a fault and analyzing the system in the case of failure. In this paper, SPN diagrams for the proposed models were implemented in the Mercury tool [24,25].

**Figure 5:**
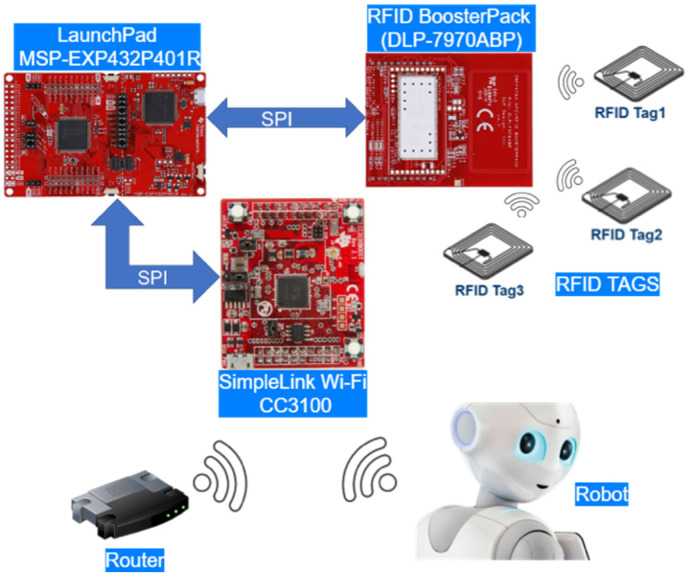
RFID object-identification system concept.

The SPN diagrams model the RFID identification process and the availability of the system in sending data to the edge layer. The concept of the modeled system is presented in Figure 5. By default, the Pepper robot cannot read RFID tags. For the proposed setup, the robot was connected to a microcontroller through a Wi-Fi link. Meanwhile, the microcontroller was responsible for controlling the RFID reader. Such a setup was needed, as Wi-Fi is the only way through which the Pepper robot can receive and send data.

The addon hardware setup is represented by Texas Instruments devices, which were configured to communicate over a serial peripheral interface (SPI):

- LaunchPad MSP-EXP432P401R—developing board based on microcontroller MSP432 (ARM Cortex M4).
- TRF7970A NFC transceiver BoosterPack (DLP-7970ABP)—NFC/RFID transceiver with 16-slot RFID frame inventory.
- CC3100BOOST—SimpleLink Wi-Fi CC3100 wireless network processor BoosterPack.

To identify objects using RFID tags, the Pepper robot must receive the tags’ UID. The challenge in RFID communication is to identify the tags through the anticollision algorithm in the shortest time possible [26]. RFID communication begins by sending a query message to which all tags in the vicinity of the RFID reader respond, resulting in a collision [27]. The purpose of the anticollision algorithm is to manage the response from each tag within the RFID reader’s range.

The implemented anticollision algorithm is specified in the ISO15693 standard [17]. According to the standard, when a collision is detected because of several tags near a reader, a mask value (*M*), mask length (*Lm*), and number of slots (*L =* 16 slots) are transmitted during the inventory command.

**Figure 6:**
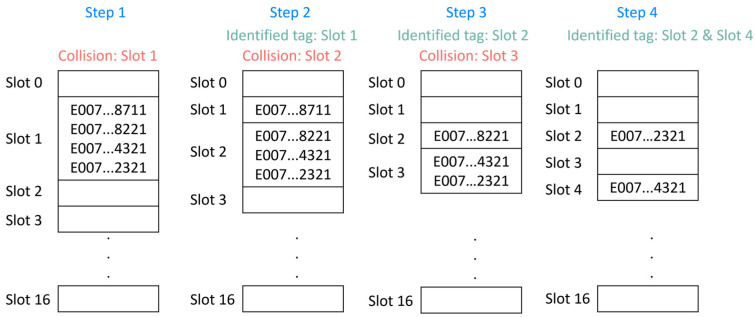
Number of steps required to solve a collision.

Upon receipt of the inventory command by the tags, they compare the least significant four bits of their UID with the associated value with the slot counter plus the mask value. Only when the two values are identical do the RFID tags respond. Although this technique helps to reduce the probability of a collision, it does not guarantee that the collision phenomenon does not occur. Figure 6 shows the anticollision process for four RFID tags. In the first step, all four tags respond to the transmission of the inventory command, generating a collision on Slot 1 of the inventory frame. To resolve the collision, it is necessary to send a new command with the mask and its associated length changed: *M* = 1 and *Lm* = 4 (bits). Via the new inventory command, the UID associated to the tag on Slot 1 is received. However, there is still a collision at Slot 2. This time, the algorithm sends a new inventory command with a new set of parameters: *M* = 2 and *Lm* = 8 (bits). The process is continued until there are no detected collisions during a frame inventory.

For the tag-identification process, three categories of slots within an inventory frame are defined:

- idle slot—a slot in the inventory frame without a response.
- single tag per slot—a slot with a single RFID tag response, the tag’s UID was received.
- collision slot—a slot in which multiple RFID tags responded. The anticollision algorithm needs to be run with a new set of values for the *M* and *Lm* parameters.

As the inquiry frame has *L =* 16 slots, the probability that a RFID tag selects a slot is equal to 1/L [18,20]. The probability that a slot is selected by multiple RFID tags is described through the following binomial distribution [18,28]:

(1) $$P_{Nt,\frac{1}{L}}\left(nts\right)=C_{Nt}^{nts}{\left(\frac{1}{L}\right)}^{nts}{\left(1-\frac{1}{L}\right)}^{Nt-nts}$$

where Nt represents the total number of tags in the range of the RFID reader, and nts represents the number of tags that select a slot for transmitting their UID.

For an RFID system with *Nt* tags, the probability that a slot is selected by one tag (i.e., nts=1) to send its UID is given by the following equation:

(2) $$P_{tag\_per\_slot}=P_{Nt,\frac{1}{L}}\left(1\right)=Nt\cdot \frac{1}{L}{\left(1-\frac{1}{L}\right)}^{Nt-1}$$

The probability of having a slot not be selected by either of the tags found in the RFID reader vicinity (i.e., *Nt*) is given by the following equation:

(3) $$P_{no\_tag\_in\_slot}=P_{Nt,\frac{1}{L}}\left(0\right)={\left(1-\frac{1}{L}\right)}^{Nt}$$

To determine the probability that multiple tags would choose the same slot, the following equation is used:

(4) $$P_{tag\_collision}=1-P_{tag\_per\_slot}-P_{no\_tag\_in\_slot}$$

**Figure 7:**
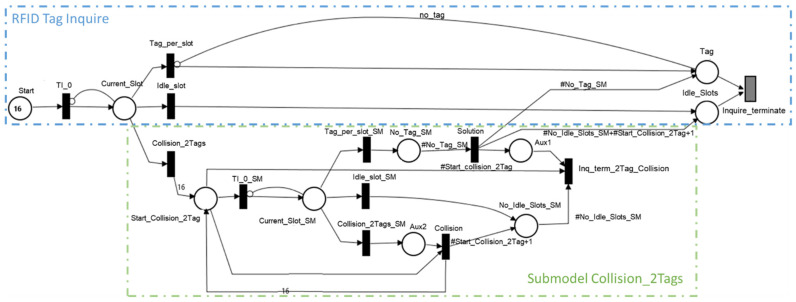
SPN RFID identification model.

Equations (2)–(4) are used in describing the RFID tag identification through an SPN model. The SPN model from Figure 7 was implemented in the Mercury tool and comprises the following components:

- Start—a place containing the number of slots that are analyzed during a frame inquiry.
- Current_Slot—the current slot indicated by the slot counter that is waiting to receive a response from the RFID tags.
- TI_0—an immediate transition that guaranties that each slot is processed one at a time. The purpose of the transition is to remove one token from Start and produce a new token in Current_Slot.
- Tag_per_slot—an immediate transition that is fired in the case that only one tag responds for the analyzed slot. This transition being fired removes the token from Current_Slot and produces one in Tag. As a result, one tag was detected from *Nt* tags present in the system.
- Idle_slot—an immediate transition that models not receiving a response during the interrogated slot.
- Collision_2Tags—an immediate transition that models a scenario in which two tags responded for the current slot. In this case, a new inquiry is needed to identify each RFID tag.

The inquiry process ends when the number of tokens in Tag is equal to the number of tags found near the RFID reader, and the entire number of slots in the frame are analyzed. To fulfil the previous conditions, a guard expression was set on immediate transition Inquire_Terminate:

(5) #Start=0

Besides the previous guard expression, the lowest priority must be set to the Inquire_Terminate immediate transition, so that the other transitions finish to fire and to avoid a conflict between two immediate transitions.

When transition Inquire_terminate is fired, Tag and Idle_Slots are depleted for all tokens. At the same time, Start is repopulated with 16 tokens representing the number of slots in a frame inquiry. This is needed to prepare the model for a new simulation. Mercury runs the model until the maximum accepted relative error between consecutive results is reached.

When designing a model with SPN, one should take care in avoiding conflicts between immediate transitions. The proposed model shows that transitions Tag_per_slot, Idle_slot, and Collision_2Tags are in such a scenario when there is a token in Current_Slot. Compared to the previous case, when we set the lowest priority to Inquire_terminate, the solution comprises setting weights for each transition [29]. The weight values represent the probability of a slot to be selected by a tag, to be an idle slot (no tag response), or multiple tags selected the slot to send their UIDs. To determine the value for the weight for each transition, Equations (2)–(4) were used. Table 1 shows the weights that are used for a system with four RFID tags.

The numbers from Table 1 show that there is a greater chance of having a collision between two tags compared to having three or all four tags in collision. As a collision implies a new inquiry frame, submodels for every scenario were created. For example, Figure 7 shows that immediate transition Collision_2Tags produces 16 tokens in Start_Slot_Collision_2Tags, which is equivalent to launching a new RFID frame inquiry. The structure of the submodel also comprises three major transitions representing the idle slot; in this case, a tag per slot or a collision between two tags. Compared to the start inquiry, when there were four tags, this time there are only two tags; the weights of each immediate transition are recomputed to reflect the collision scenario.

Compared to other studies, through the SPN model, we change the conditions of each inquiry frame. By firing the transitions millions of times, we can determine the probability of having different events (i.e., one tag per slot, idle slots, and pairs of tags in collision). Changing the probabilities of each of the three major transitions depending on the number of tags that need to be identified results in an improved probability estimation of the number of frames needed to identify the tags, and thereby the time needed to identify the objects.

### 2.2. SPN Submodels to Analyze Collision between RFID Tags

**Figure 8:**
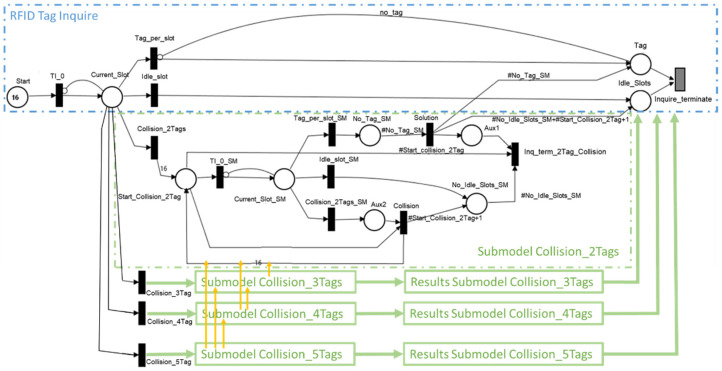
SPN RFID identification submodel structure.

The proposed model comprises submodules capable of solving a collision among two, three, four, and five RFID tags (Figure 8). The submodel analyzes the collision among a smaller number of tags by using a new set of weights for an idle slot, single tag per slot, and collision-type slots. A submodel can access other submodels that resolve collisions among a smaller number of tags. This approach allows for estimating the probability of needing a new frame to solve a collision.

**Figure 9:**
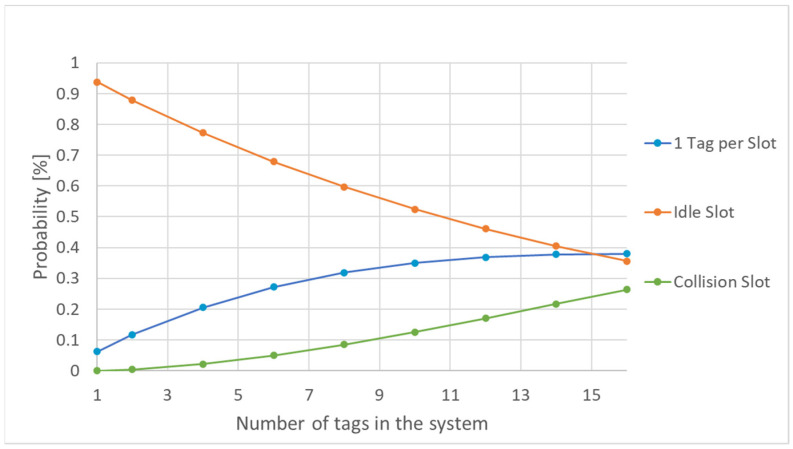
Slot-type probability vs. number of tags in the system.

As the number of tags increases towards available system slots, so does the probability among the three types of transitions shifts (Figure 9). Therefore, submodel implementation allows for determining an estimation of the number of frames needed in identifying the RFID tags’ UID.

A submodel implementation relays on the values provided by Equations (2)–(4). Each submodel has places that monitor the number of identified tags (i.e., one tag per slot), idle slots, or the types of collisions that occurred. In the end, each triggered submodel populates Tag with the number of identified tags (Figure 7).

From the main model, a collision of three tags is triggered. The designated submodel identifies one tag and launches the submodel that deals with the collision between two RFID tags. As a result, the places are populated as follows:

- the main model populates Idle_Slots with 15 tokens, plus one collision slot;
- the submodel dealing with the collision among three tags populates Tag with one token and Idle_Slots with 14 tokens (i.e., one inquiry frame), plus one collision slot;
- the submodel dealing with the collision between two tags populates Tag with two token and Idle_Slots with 14 tokens.

For the previous example, a number of three inquiring frames (48 analyzed slots) are needed to receive the tags’ UID. However, the number of tokens in Idle varies in case the collision among the three tags extends over a larger number of bits, a scenario that can also apply to a collision between two RFID tags.

Setting the weight value for the immediate transition model (i.e., Tag_per_slot, Idle_slot, Collision_xTags) solves the conflict between the transitions until a point in the RFID inventory. During this process, the system needs to parse all the RFID tags and know their status: identified or in a collision. Therefore, as the slots are processed and there are unidentified tags, the Idle_slot transition needs to be deactivated through the following guard expression:

(6) (#Start+#Current_Slot)≥2 OR ( #Tag=nts)

When all tags are identified, transition Idle_slot is kept active. However, if there is one remaining slot to process and there are unidentified tags, their status should be identified or in a collision.

As a result, guard expressions are also needed for immediate transitions Tag_per_slot and Collision_xTags (*x* represents the number of tags involved in a collision). Immediate transition Tag_per_slot is invalidated through the inhibitor arc from Tag. When the number of tags in Tag are equal to *nts*, Tag_per_slot is deactivated. For Collision_xTags, the following guard expression was implemented:

(7) ((#Tag ≤nts−x) AND (#Start+#Current_Slot)≥2 ) OR ((#Start+#Current_Slot)=1 AND (nts−#Tag)=2 ),

where x represents the number of tags involved in a collision.

Guard expression (7) comprises two sections. The first section is represented by the expression:

(8) ((#Tag ≤nts−x) AND (#Start+#Current_Slot)≥2 ),

which verifies that, in the vicinity of the RFID reader, there are a number of tags at least equal to the one that could cause the collision analyzed by the submodel. It is necessary to make sure that there are at least two more slots to analyze if the number of tags left for identification is higher than the number of tags involved in the collision process.

The second section of the monitoring condition,

(9) ((#Start+#Current_Slot)=1 AND (nts−#Tag)=2 ),

Ensures that, when there is only one slot left to be analyzed and unidentified tags, the submodel dealing with the collision between the remaining tags is executed.

The SPN model was stopped at analyzing five RFID tags, as the submodels’ implementations were becoming very difficult to organize in Mercury. One disadvantage of SPN modeling consists in not being able to call the submodels as functions are in programing language. As an alternative solution, a MATLAB implementation was developed on the basis of equations and the SPN approach to model the FSA anticollision protocol.

### 2.3. RFID Collision Analysis through MATLAB

**Figure 10:**
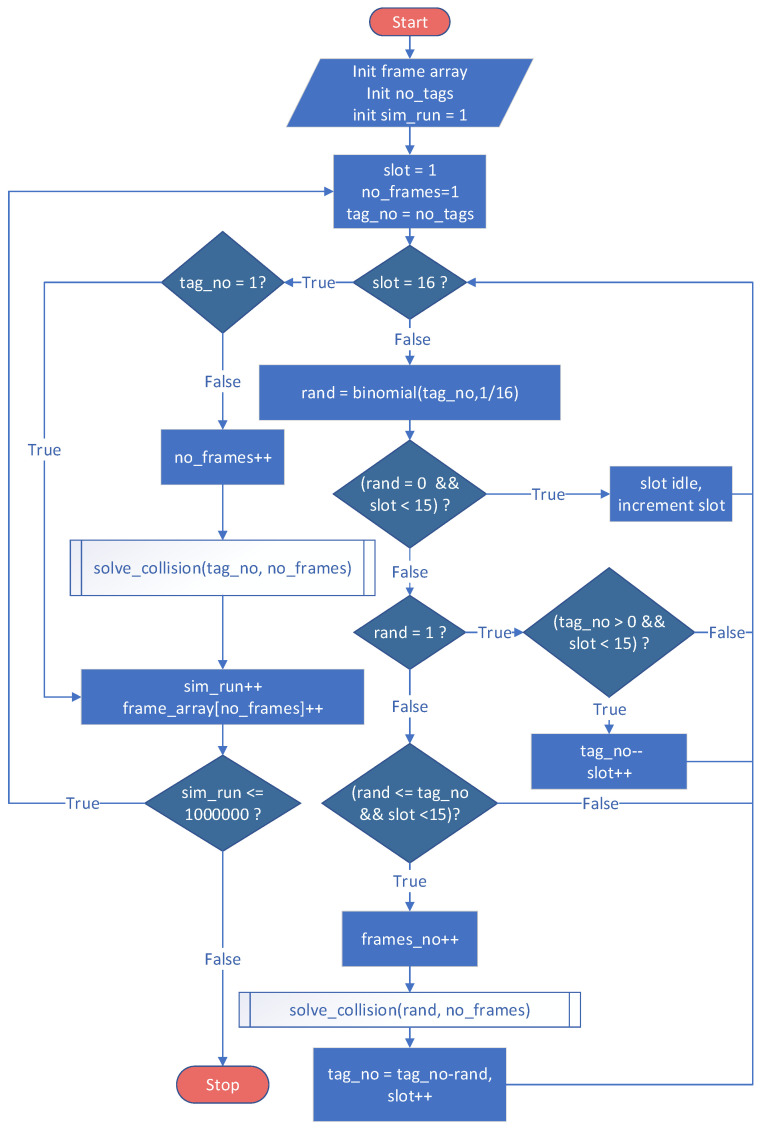
MATLAB RFID identification model.

The MATLAB model has the same approach as the one proposed in Mercury, except that the transitions were replaced with the switch instruction. The probability of choosing a certain branch is obtained through a binomial distribution function. The parameters of the binomial function are determined on the basis of the number of tags near the RFID reader. As in SPN implementation, a record of tags involved in collisions was kept. Through this approach, collisions between tags from different groups were avoided. Figure 10 illustrates the software diagram used in implementing the model in MATLAB. Each scenario, which is defined through the number of slots in a frame and the number of tags in the vicinity of the RFID reader, was run 106 times to obtain the probability data.

The implemented models in Mercury and MATLAB allowed for determining the probabilities associated with the identification of RFID tags in the vicinity of an RFID reader in a certain number of frames. In the literature, emphasis is on reaching maximal throughput (number of identified RFID tags vs. time needed to identify them). The aim of this study was to find the optimal number of tags that could be identified in the shortest possible identification time and with the highest possible probability of achievement. To compare the obtained results through the simulations, a cost function is proposed:

(10) Cost=nf·t_frameNt [timetag],

where

- nf—number of frames needed to solve the collision between tags and obtain their UID;
- t_frame—time length of a frame inventory (implementation-dependent);
- *Nt*—number of tags in the RFID reader vicinity.

For example, if the identification of seven tags within a system has an associated time of 2·t_frame, the cost of the detection of a tag is:

(11) Cost=2·t_frame7=0.29 [t_frametag]

The above equation was defined for a general case. Depending on the implementation, the duration of *t_frame* may vary. However, the principle of defining the cost remains unchanged.

The proposed model allows for the identification of the optimal number of tags (identified object), while considering the identification time and the probability of occurrence. By introducing the cost function, this process becomes identifying the lowest cost that has the highest possible associated occurrence probability. Having the number of tags equal to the number of slots in a frame yields the highest throughput (i.e., the smallest cost) if all tags are read in one frame [18,20,28]. As Section 3 shows, such a scenario has a small probability of occurrence, but there is also large variation in the time needed to identify all tags in the system.

### 2.4. RFID Object Identification at the Edge—Availability

**Figure 11:**
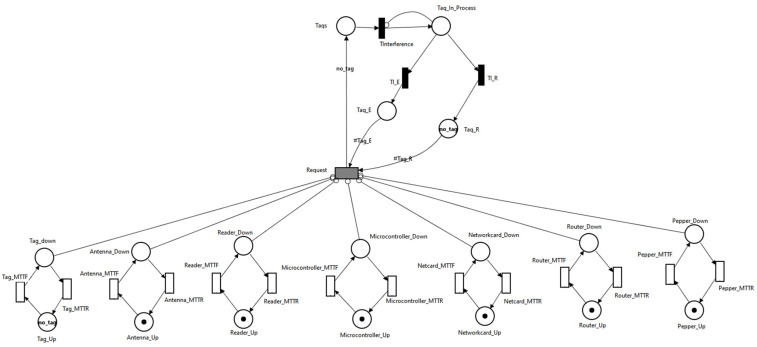
SPN model for object-identification system.

To model the system produced by an RFID hardware addon, Pepper robot, and edge router (Figure 5), the SPN model from Figure 11 was created.

Modeling the proposed scenario through SPN involved:

- modeling the erroneous reception of associated bits with the UID of a tag by Tag_E (tags whose UID was incorrectly received) and Tag_R (tags whose UID was correctly received);
- modeling the operation of RFID tags through Tag_Up and Tag_Down locations;
- modeling the operation of the RFID antenna through the Antenna_Up and Antenna_Down locations;
- modeling the operation of the RFID reader through the Reader_Up and Reader_Down locations;
- modeling the operation of the microcontroller through the Microcontroller_Up and Microcontroller_Down locations;
- modeling the operation of the network card through the Networkcard_Up and Networkcard_Down locations;
- modeling the operation of the router through the Router_Up and Router_Down locations; and
- modeling the operation of the Pepper robot through the Pepper_Up and Pepper_Down locations.

The modeling of the erroneous reception of bits associated with the UID of a tag through SPN was performed by setting weights for immediate transitions Tag_E and Tag_R. To calculate the required weights for the model, one must understand the process by which reception errors occur. If on–off keying (OOK) modulation is used, the probability that a bit is correctly received is described by the following equation [30]:

(12) $$P_{bit\_corect}=1-\frac{1}{2}\cdot e^{-\frac{E_{b}}{2N_{\sigma }}},$$

where $\frac{E_{b}}{2N_{\sigma }}$ is the signal-to-noise ratio.

The used tags are from Texas Instruments and have the UID encoded through 64 bits [31,32]. Thus, the probability of erroneously receiving the UID associated with a tag is given by the equation:

(13) $$P_{tag\_UID\_eronat}=1-P_{bit\_corect}{}^{N},$$

where *N* represents the number of bits used in encoding the UID. The probability of receiving the UID is 93.80%, while that of erroneous reception is 6.20%. The values mentioned above passed as weights for immediate transitions Tag_R and Tag_E, respectively.

To determine the availability of the system described in Figure 11, the following metrics were used:

- determining the availability when all the tags are correctly identified, there are no errors in receiving UIDs:

(14) P{(#Tag_R=Nt)};

- determining the availability when, at most, one UID code is received erroneously:

(15) P{(#Tag_R≥Nt−1)};

- determining the availability when, at most, two UIDs are erroneous:

(16) P{(#Tag_R≥Nt−2)}.

**Figure 12:**
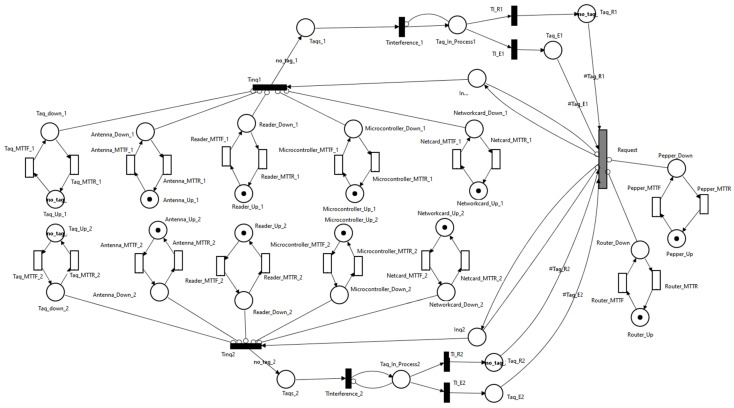
SPN model for object-identification system—second scenario.

**Figure 13:**
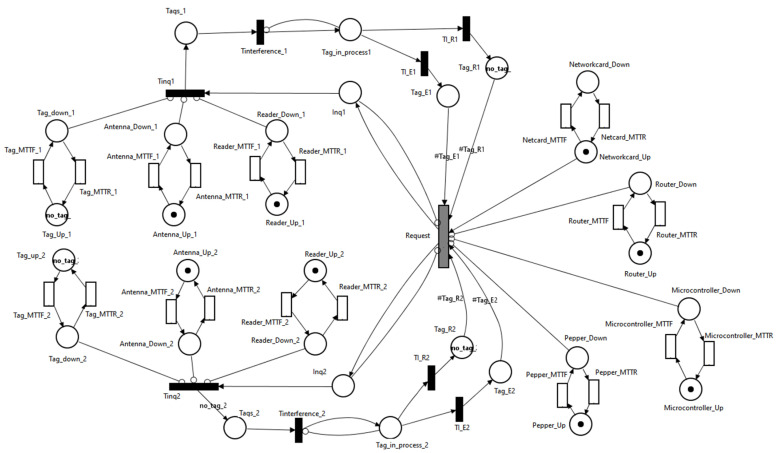
SPN model for object-identification system—third scenario.

The scenario shown in Figure 11 represents the situation in which the Pepper robot has the RFID addon attached. Besides this scenario, two other scenarios were analyzed; this time, the RFID addon was no longer attached to Pepper robot:

- Pepper, multiple (Wi-Fi + RFID reader) modules scenario in which the Pepper robot communicates with multiple Wi-Fi + RFID reader modules (Figure 12). Each module is responsible for monitoring the RFID tags.

- Pepper, Wi-Fi, multiple RFID reader: In this scenario, there was a single microcontroller with Wi-Fi capabilities that acted as an intermediate point between the Pepper robot and RFID readers (Figure 13). The microcontroller stored the UID of each tag found near the RFID readers.

To compare the availability of the three scenarios, an equal number of tags were used in the system:

- Scenario I: *Nt* tags are in the system;
- Scenario II: *Nt* tags are in the system, the first pair of Wi-Fi + RFID reader deals with *Nt/2* tags, and the other half is read by the second pair of Wi-Fi + RFID reader;
- Scenario III: *Nt* tags are in the system; the first RFID reader deals with *Nt*/2 tags, the other half is read by the second RFID reader.

The metrics of Scenarios II and III were implemented, such that the availability results of the system could be compared with those obtained in the first scenario:

- determining the availability when all tags are correctly identified, there are no errors in receiving UIDs:

(17) P{(#Tag_R1=Nt/2) AND (#Tag_R2=Nt/2)};

- determining the availability when one UID code at most is received erroneously:

(18) P{( (#Tag_R1≥Nt/2−1) AND (#Tag_R2=Nt/2) ) OR ( (#Tag_R1=Nt/2) AND (#Tag_R2≥Nt/2−1) )};

- determining the availability when two UIDs at most are erroneous:

(19) P{( (#Tag_R1≥Nt/2−1) AND (#Tag_R2≥Nt/2−1) ) OR ( (#Tag_R1=Nt/2) AND (#Tag_R2≥Nt/2−2) )OR ( (#Tag_R1=Nt/2−2) AND (#Tag_R2≥Nt/2) )}.

**Figure 14:**
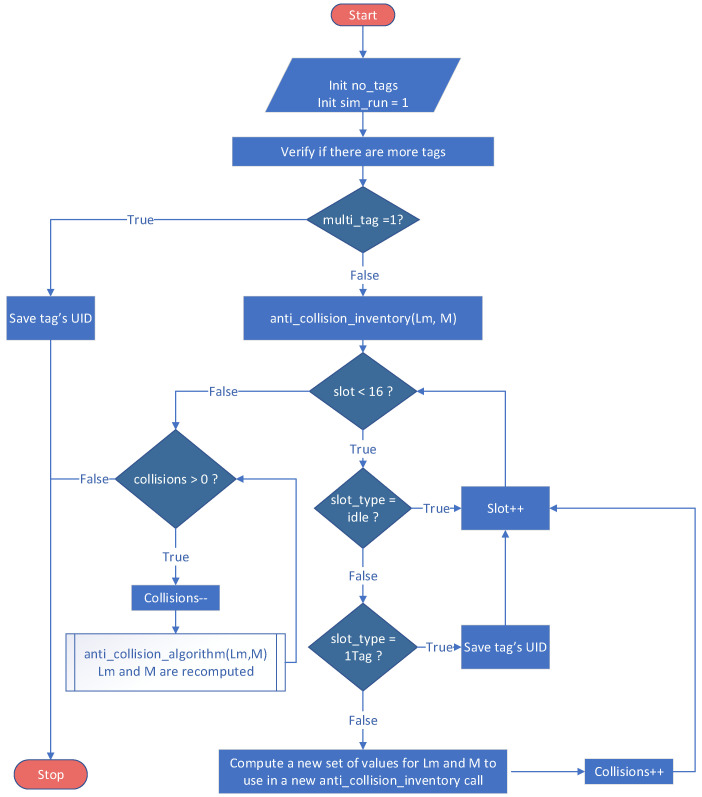
ISO/IEC 15693 implementation software diagram.

The first scenario modeled through SPN in Mercury was also physically implemented by using the hardware mentioned in Figure 5. LaunchPad MSP-EXP432P401R configures and transmits messages between two wireless communication modules. Regarding RFID communication, the ISO/IEC 15693 standard algorithm was implemented. Recursive implementation was performed, which allows for storing a tag’s ID in a buffer as it is identified as a single answer on the query slot (Figure 14).

When identifying a collision, the length and value of the mask that is used in the next inventory frame are calculated. The computed pair is placed in a queue, as there is the possibility of multiple collisions within a frame.

This aspect encountered in practice is also mimicked in submodel implementation through the monitoring expressions described in Equations (6) and (7). The communication between Pepper and the RFID module is via the CC3100BOOST Wi-Fi module. Thus, it was necessary to implement a TCP-IP WebSocket server on the MSP432 microcontroller for server-client communication. The steps performed by the application were to obtain an IP within the local network, and then to initialize the server by opening a socket to receive messages from the client.

The client application runs on the Pepper robot, and it was implemented in the Python programming language and transposed into the graphics blocks used in Choregraphe. For the analyzed scenarios, the Pepper robot knew the IP addresses assigned to servers used in communication with the RFID transceivers. The client application sends the inventory command and then receives the list of UIDs corresponding to tags found in the vicinity of the transceiver.

## 3. Results

### 3.1. RFID Tag Detection as a Function of Cost

**Figure 15:**
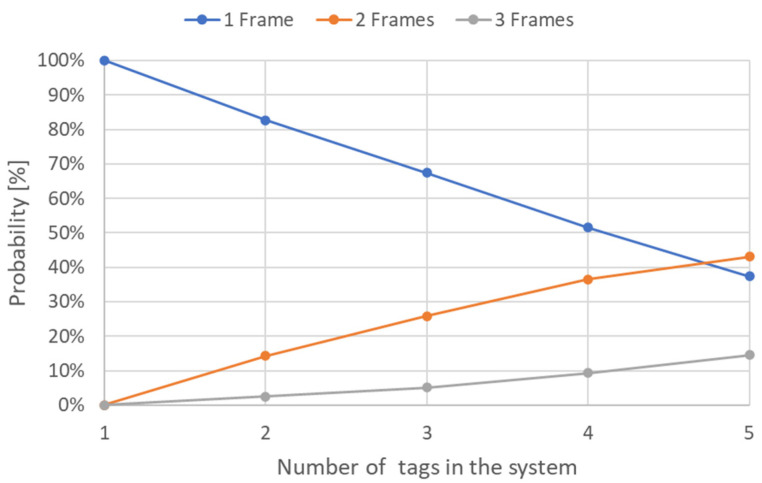
Number of frame probability distribution vs. number of tags in the system.

The first type of analysis performed over the presented models showed the relationship between the number of frames that might be needed to identify the RFID tags versus the number of tags in the system (Figure 15). Results from Figure 15 were obtained by performing an SPN stationary simulation over the model from Figure 8 within the following parameters:

- confidence level of 95%;
- maximal relative error of 3%;
- batch size of 10,000;
- *Nt* domain was set to [1,5].

**Figure 16:**
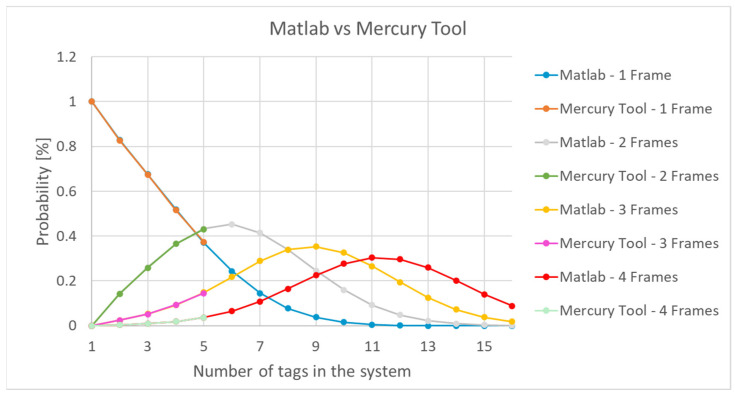
Frame probability distribution vs. number of tags in the system—MATLAB implementation.

Analyzing the values in Figure 15 showed that, from a number greater than 5 tags, the probability of detecting the tags in at least two frames was higher than the probability that they would be detected by a single frame. By using the MATLAB model implementation, the impact of several tags could be observed (Figure 16).

**Figure 17:**
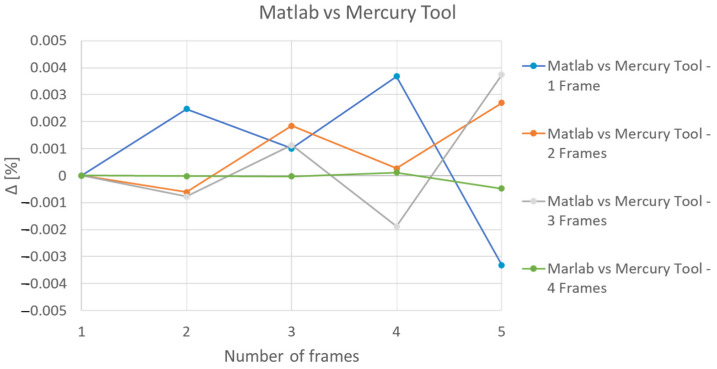
Deviations: MATLAB vs. Mercury model.

Figure 16 shows a comparison of the results with those obtained in Mercury. The deviation between the two implementations was below 0.4% (Figure 17).

**Figure 18:**
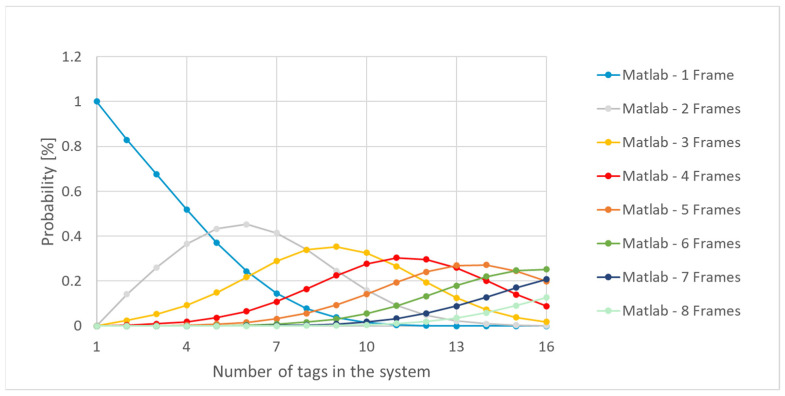
Number of frame probability distribution vs. number of tags in the system—MATLAB model up to 8 frames.

The implementation in MATLAB shows the distribution of probabilities associated with the number of frames needed to detect system RFID tags (Figure 18). As the system approached maximal capacity, the probability of identifying tags was distributed among scenarios that required multiple frames to solve the tag identification process.

Figure 18 shows that the probability of finding the UIDs of a number of 16 tags through five frames was equal to the probability of completing the identification process after seven frames, which was translated into a time uncertainty of 2·t_frame.

**Figure 19:**
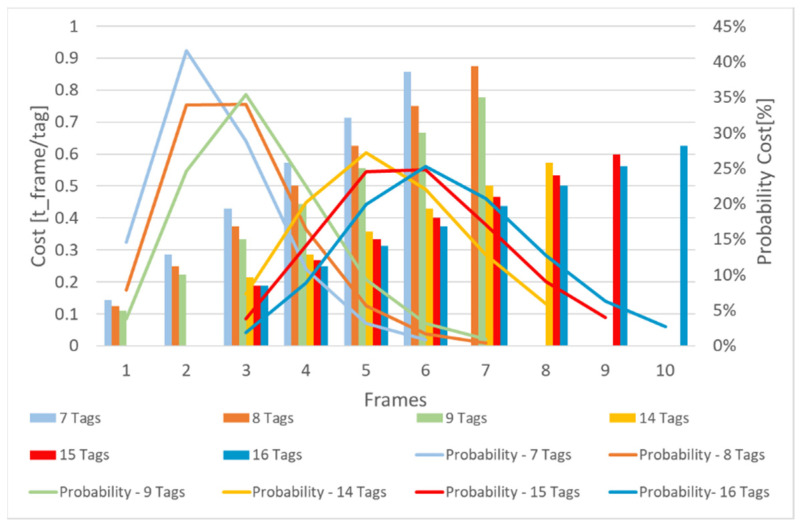
Cost value vs. cost probability.

Figure 19 shows the costs, according to Equation (10), related to the identification of the tags for a system with 7, 8, and 9, and 14, 15, and 16 tags, respectively. In addition, the probability of reaching identified costs during the operation of the system is represented. These were obtained from the analysis implemented in MATLAB.

The costs of identifying 14, 15, or 16 tags were much lower compared to identification costs of 7, 8, or 9 tags. The costs associated to 14, 15, or 16 tags remained below the values of the other scenarios (i.e., 7, 8, or 9 tags), even if the number of frames needed for identification increased (Figure 19).

Even if the cost function showed a favorable operating situation at full system capacity, considering the probability with which the costs occur, scenarios could be identified in which the costs are lower. For example, in the scenario with 16 tags near the RFID reader, the most likely costs are those associated with a number of 5, 6, 7 frames, corresponding to a cost of 0.31, 0.38, and 0.44 t_frametag, and having an occurrence probability of 19.91%, 25.21%, and 20.76%, respectively.

Closer analysis shows that the associated cost for five frames can manifest itself with the same probability as that for the one with seven frames. The costs associated with the analyzed cases represent a total probability of occurrence of 65.88%.

If there are only eight tags in the system’s vicinity, the scenarios with the highest probabilities are those with two and three frames, characterized by a cost of 0.25  and 0.38 t_frametag, respectively, and having an occurrence probability of 33.90% and 33.95%, respectively. In this situation, the cost is maintained in a smaller interval, which represents 67.85% of the possible situations.

For a scenario comprising seven tags, detection is most often performed through two or three frames, having a cost of 0.29 t_frametag with a probability occurrence of 41.52%, and 0.43 t_frametag with an associated probability of 28.87%, respectively.

From another perspective, there are situations where, for the same number of tags near the RFID reader, a smaller number of frames have the same probability of occurrence as that of scenarios characterized by a higher number of frames (Figure 18). Such a scenario is observed if we have 14 tags to identify. For a system with 14 tags, the results of Figure 18 show the same probability for identifying the tags through a three-frame scenario as through eight frames. In such a situation, the system response would be characterized by large time delays, as the two scenarios have the same occurrence-probability value.

On the basis of results from Figure 18 and Figure 19 to have a system with a constant response time, the total number of slots in the system must be almost double the number of tags that are near the RFID reader.

### 3.2. RFID Object-Detection System Availability

By implementing the three RFID object-identification system scenarios in Mercury via SPN, the availability of the systems was determined using the metrics described by Equations (14)–(19) (Table 2). Although Scenarios 2 and 3 contain several devices, which would suggest a higher failure occurrence rate, the differences in availability compared to that in Scenario 1 are very small. This is because of the high values, of the order of thousands of hours, for the MTTF parameter associated with the devices used in reading and identifying RFID tags [22]. Results from Table 2 show that there is more to an RFID object-detection system than the throughput (number of identified tags in a short amount of time). Due to the radio interference, there is also the probability that a tag UID is received erroneously.

As the number of tags in the system increases, the probability of receiving an erroneous tag also rises. Results from Table 2 prove that, because of the bit error, the probability of correctly receiving all identification codes (which is needed when maximal throughput is desired) is around 35%. However, decreasing the number of tags also diminishes the chance of having tags affected by electromagnetic interferences.

Coupling the availability of the system with the cost function allows for identifying the optimal point of operation for an RFID object-detection system: the maximal number of tags with a predictable time detection that offers the highest availability.

To verify the SPN model results, the first scenario was physically implemented. After running the proposed scenario for 1168 h, the following events were recorded:

- 11 events in which, out of four tags, only three were read;
- Three events required resetting the system, and there were problems in transmitting messages that led to the system being blocked;
- Three events occurred in which the sockets were not closed correctly, resulting in the loss of messages with the server.

Following analysis of the logs on both the server and those associated with the Pepper robot, the availability of the system was determined. For the MTTR parameter associated with the Pepper robot, the value of 0.96 h was used [22], and for the MTBF, a value of 68.71 h was calculated. On the basis of obtained values, an availability of 98.6219% was determined. The value is close to the result obtained through the SPN for the scenario in which at least one erroneous tag is accepted.

## 4. Conclusions

Our SPN model and submodel implementation allow for the recalculation of probabilities in terms of events (tag per slot, idle slot, or collision slot) depending on the number of tags that generated the collision. This proposed model approach follows the steps in ISO/IEC 15693 to extend the length of the mask and its associated value at the time of a collision.

Compared to the analysis proposed in [22], when running a collision inquiry frame, the condition of identifying at least one tag is no longer required. Therefore, running a collision inquiry frame could result in a new collision handling frame because tag UIDs have identical values on a larger number of bits. The new model and the new set of monitoring conditions resulted in a decrease in probabilities depending on the number of tags for single-frame detection by approximately 20%. At the same time, the probabilities associated with multiframe detection increase significantly, denoting a greater impact of two- and three-frame detection, respectively. Compared to other studies [18,19,20,21], we proved that an FSA anticollision algorithm achieving maximal throughput (i.e., each frame slot occupied by a single tag) has a low probability.

Such a result greatly impacts the timings of a system that aims to identify objects by RFID. To determine the optimal configuration between the number of tags served by an RFID reader and the time in which it would read them, a cost function was proposed. The cost analyses identified scenarios with a fast response time versus the number of identified RFID tags. This is especially important for real-time applications that are based on receiving data at well-predefined time intervals.

There are no major differences between the three proposed scenarios in terms of availability, because the duplicated components (i.e., microcontroller, RFID chips) are characterized by the MTTF parameter with values of thousands or even millions of hours. By combining the cost function with the availability of the system, we determined the optimal point in which the maximal number of tags with predictable time detection that offered the highest availability can be achieved: the number of slots in a frame should be double than the RFID tags in the system.

The analyzed scenarios also proved that systems with a larger number of RFID tags needing to be identified can be created. By increasing the number of RFID antennas or readers, the tag identification time is maintained, while system availability is not affected.

During physical implementation, most of the situations that affected system availability were caused by software applications associated with the server and client application.

The RFID object-identification system can be seen as a complementary component for a system based on advanced image processing (e.g., person identification, medication administration, context build). In a cloud–fog–edge architecture, the two components can be precisely shared on different layers to counteract problems, such as response time or data bandwidth, which may be encountered at the cloud-computing level. The advanced image-based identification and processing component and context analysis algorithms can be processed at the cloud and fog levels; at the edge layer, RFID technology is suitable. The advantages of RFID technology primarily consist of low power consumption and low processing requirements, which are aspects compatible with IoT devices that are served by this level.

## Figures and Tables

**Table 1:** Immediate transition probabilities for handling slots within an identification frame: 4 tags and a 16-slot frame.

Slot Type	$P_{Nt,\frac{1}{L}}\left(nts\right)$	Probability of Occurrence
One tag per slot	$P_{tag\_per\_slot}=P_{4,\frac{1}{16}}\left(1\right)$	20.5993652%
Idle slot	$P_{no\_tag\_in\_slot}=P_{4,\frac{1}{16}}\left(0\right)$	77.2476196%
Slot with collision	$1-P_{tag\_per\_slot}-P_{no\_tag\_in\_slot}$	2.1530151%
Slot with 2-tag collision	$P_{2tag\_collision}=P_{4,\frac{1}{16}}\left(2\right)$	2.0599365%
Slot with 3-tag collision	$P_{3tag\_collision}=P_{4,\frac{1}{16}}\left(3\right)$	0.0915527%
Slot with 4-tag collision	$P_{4tag\_collision}=P_{4,\frac{1}{16}}\left(4\right)$	0.0015259%

**Table 2:** Availability of analyzed scenarios vs. number of tags in the system.

No. of Tags	Scenario 1			Scenario 2			Scenario 3		
	No Errors	One Erroneous Tag	Two Erroneous Tags	No Errors	One Erroneous Tag	Two Erroneous Tags	No Errors	One Erroneous Tag	Two Erroneous Tags
2	87.9900%	99.6062%	N/A	87.9569%	99.6212%	N/A	88.0242%	99.6423%	N/A
4	77.5855%	97.9709%	99.9000%	77.3842%	97.8143%	99.8960%	77.1889%	97.8651%	99.8988%
6	68.1615%	95.1877%	99.5746%	68.0436%	94.9642%	99.5199%	67.9565%	95.1738%	99.5216%
8	60.5690%	91.8783%	98.8528%	59.8911%	91.3952%	98.9691%	59.7986%	91.5098%	98.9798%
10	52.3894%	87.4789%	97.7052%	52.3087%	87.3695%	97.9219%	52.9554%	87.6973%	97.9450%
12	45.7768%	82.9385%	96.4815%	46.3120%	83.2289%	96.5695%	46.3120%	83.2031%	96.5885%
14	41.0236%	78.6182%	95.0974%	40.8684%	78.5720%	94.8635%	40.7682%	78.5771%	94.9531%
16	35.5921%	73.6819%	92.5009%	36.1016%	73.6395%	92.6258%	35.7017%	73.6912%	92.5994%

## Data Availability

Not applicable.
